# Supplementary figures and images for: Imprinted Dlk1 dosage as a size determinant of the mammalian pituitary gland
Source: eLife. 2023 Aug 17;12:e84092. doi: 10.7554/eLife.84092 (PMC10468206; doi:10.7554/eLife.84092)

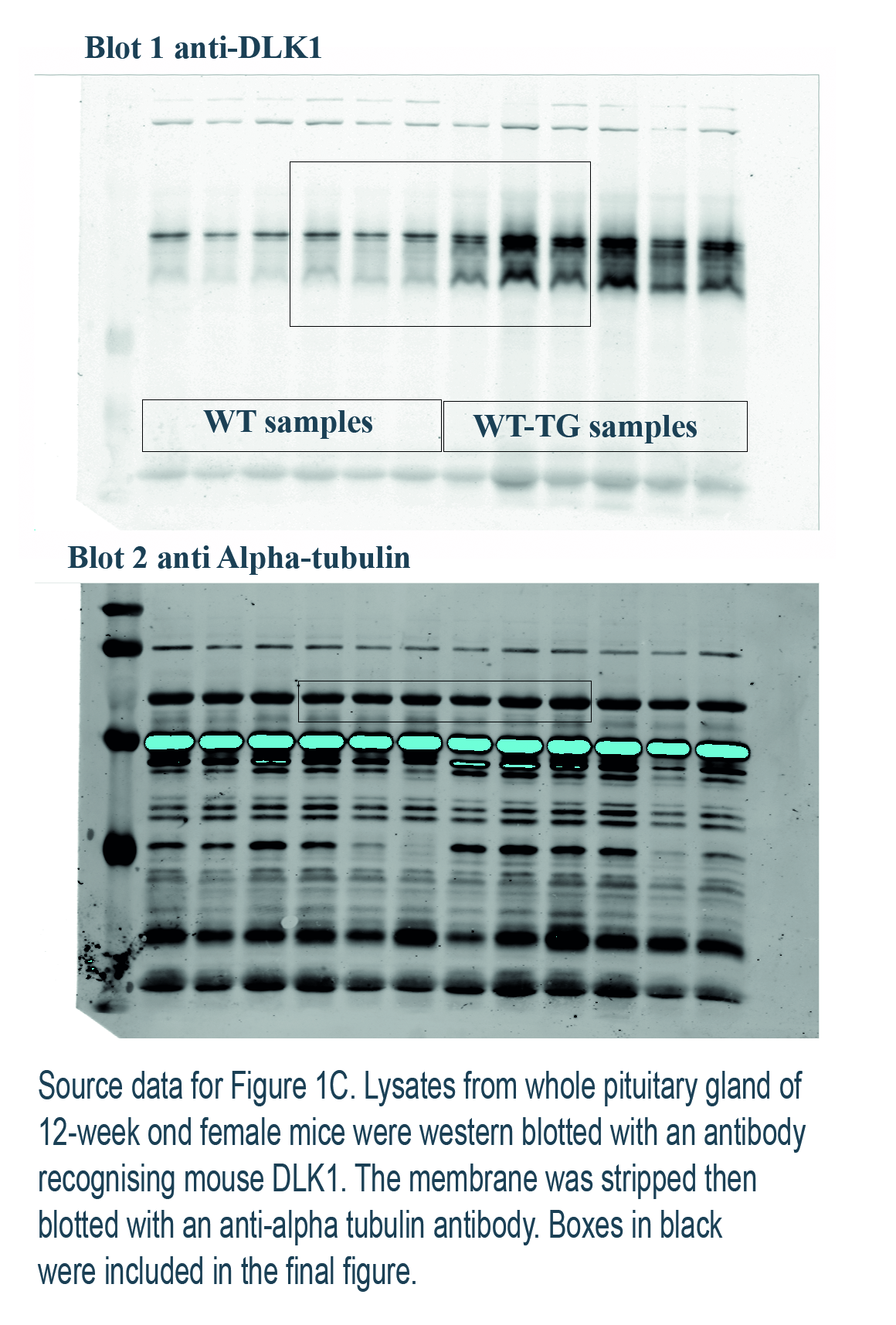

Supplement: Figure 1—source data 2. [file elife-84092-fig1-data2.zip › Source_data_gels_Figure1C/Source_data_Fig1C.tif]

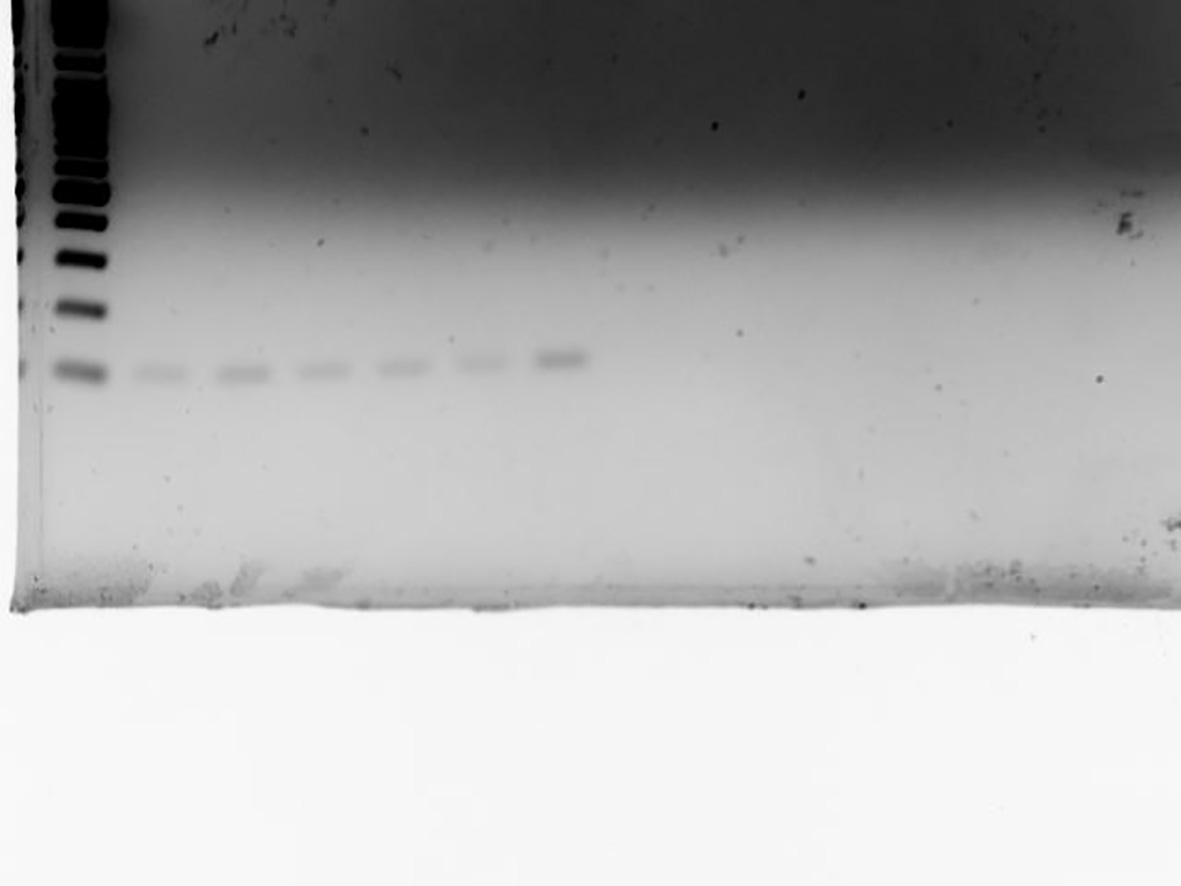

Supplement: Figure 2—source data 1. [file elife-84092-fig2-data1.zip › Source_data_Figure_2D/aTub PCR.jpg]

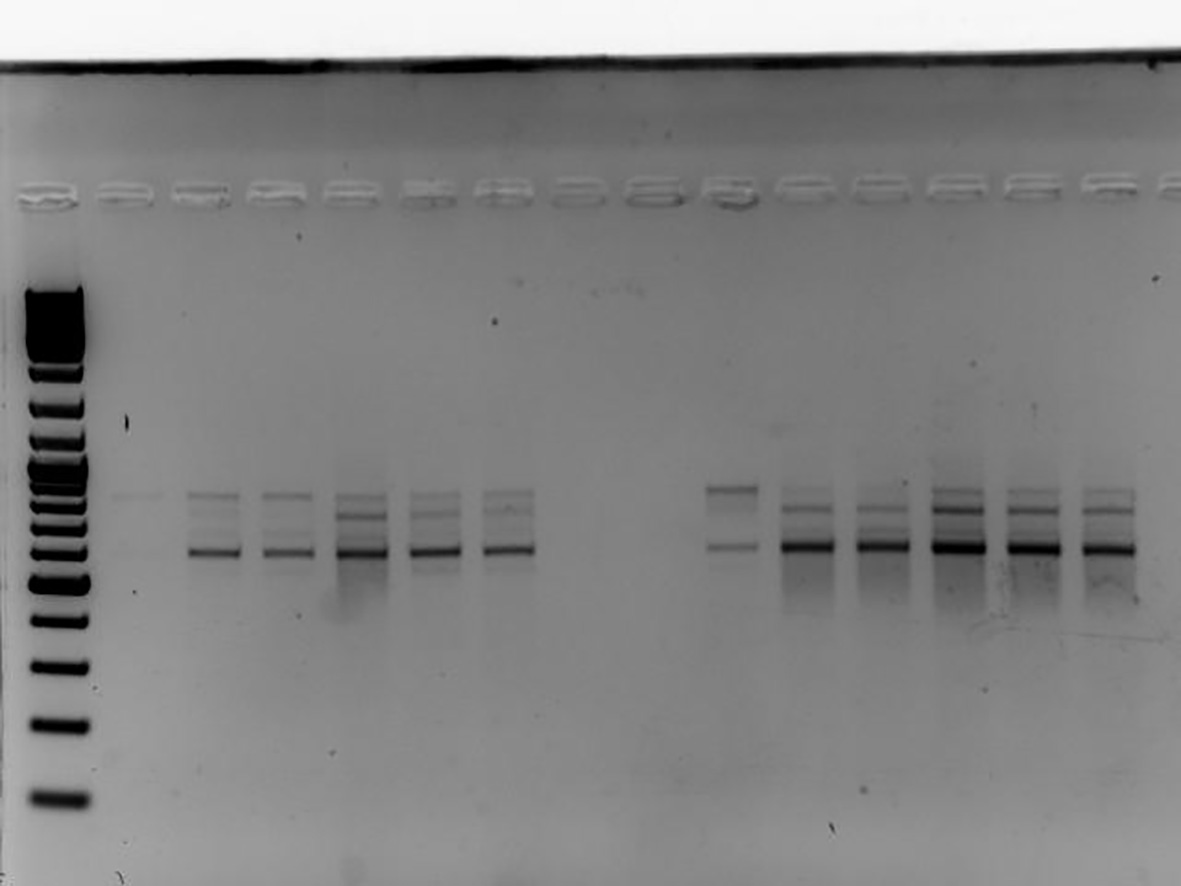

Supplement: Figure 2—source data 1. [file elife-84092-fig2-data1.zip › Source_data_Figure_2D/Dlk1 PCR.jpg]

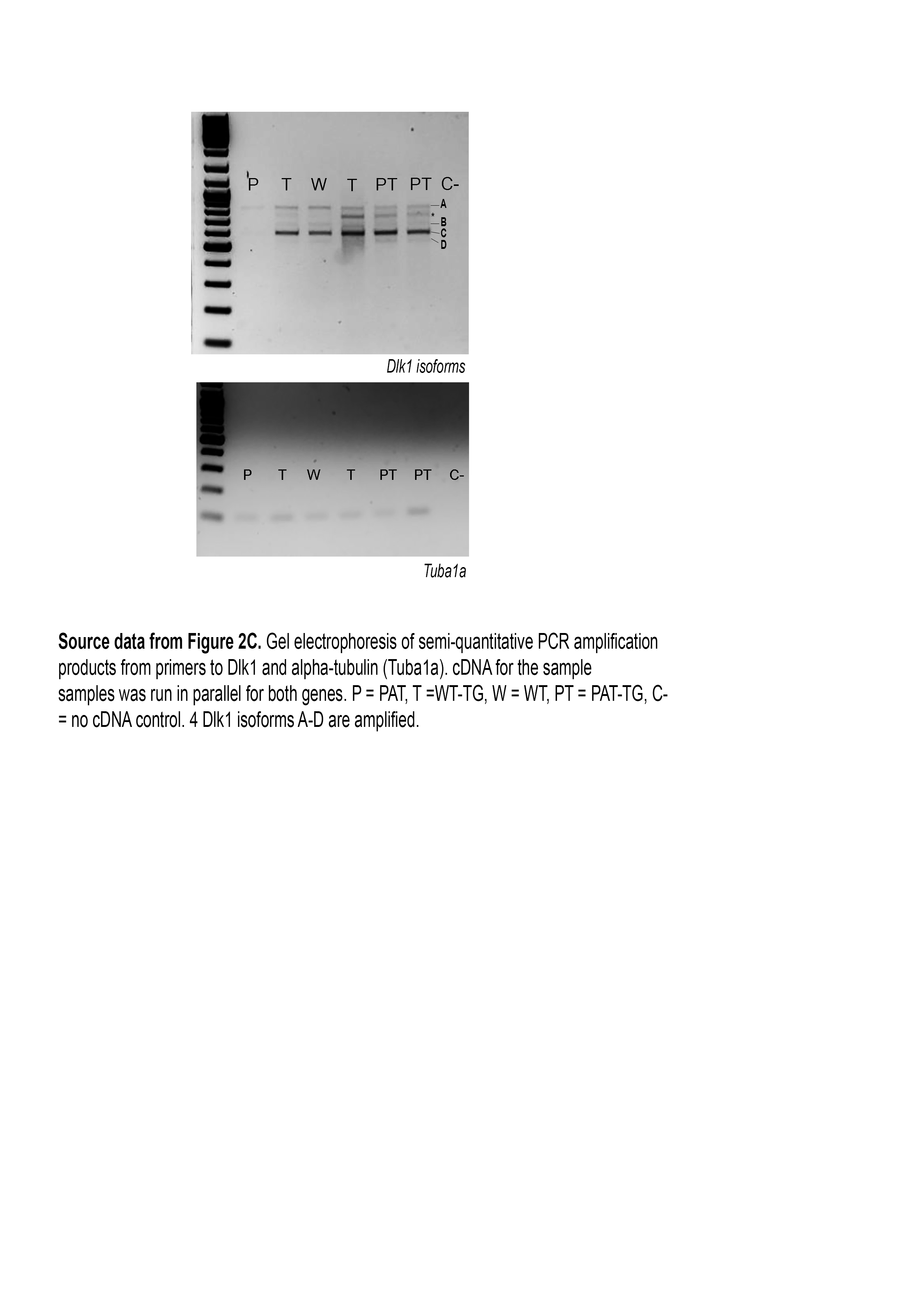

Supplement: Figure 2—source data 1. [file elife-84092-fig2-data1.zip › Source_data_Figure_2D/Source_data_Fig2D.tif]
