# Supplementary material for: Imprinted Dlk1 dosage as a size determinant of the mammalian pituitary gland
Source: eLife. 2023 Aug 17;12:e84092. doi: 10.7554/eLife.84092 (PMC10468206; doi:10.7554/eLife.84092)
Supplement: Supplementary file 1. — (a) Proportions of AP cells labelled with hormonal markers in WT and WT-TG female animals at 12 weeks of age. (b) Total body mass of animals from matched litters sacrificed from E11.5 to P21. Individuals in each age group were compared by One-Way ANOVA with post-hoc pairwise testing WT vs WT-TG, PAT, PAT-TG and PAT vs PAT-TG, corrected for multiple comparisons using Bonferroni’s adjustment. (c) Pituitary volumes acquired by stereological estimation in the embryo. Data from males and females is combined. (d) Pituitary volumes acquired by stereological estimation in of the intact postnatal gland. Individuals in each age group were compared by One-Way ANOVA with post-hoc pairwise testing WT vs WT-TG, PAT, PAT-TG and PAT vs PAT-TG, corrected for multiple comparisons using Bonferroni’s adjustment. Data from males and females is combined. (e) Pituitary volumes acquired by stereological estimation in of the intact adult gland. All animals were compared by Two-Way ANOVA with post-hoc pairwise testing WT vs WT-TG, PAT, PAT-TG and PAT vs PAT-TG, using Dunnett’s multiple comparison test. (f) Proportion of proliferating cells (IHC positive for p-HH3) in the embryonic and postnatal pituitary gland. Data from males and females is combined. (g) RNAScope probes used in this study. (h) Primary antibodies used in the study. (i) RT-PCR and RT-qPCR primers used in this study. [file elife-84092-supp1.docx]

## SupplementaryTables

|  |  | **WT** | | | **WT-TG** | | |
| --- | --- | --- | --- | --- | --- | --- | --- |
| *Cell* | *Subgroup* | *Mean* | *SD* | *n* | *Mean* | *SD* | *n* |
| GH+ | **Total proportion** | **0.290** | **0.023** | 7 | **0.294** | **0.020** | 7 |
|  | DLK1-/GH+ | 0.019 | 0.003 |  | 0.014 | 0.002 |  |
|  | DLK1+/GH+ | 0.271 | 0.008 |  | 0.280 | 0.007 |  |
|  | *% co-stained DLK1* | *93* |  |  | *95* |  |  |
| PRL+ | **Total proportion** | **0.213** | **0.012** | 4 | **0.201** | **0.018** | 4 |
|  | DLK1-/PRL+ | 0.135 | 0.003 |  | 0.130 | 0.011 |  |
|  | DLK1+/PRL+ | 0.078 | 0.004 |  | 0.072 | 0.002 |  |
|  | *% co-stained DLK1* | *37* |  |  | *36* |  |  |
| TSH+ | **Total proportion** | **0.096** | **0.011** | 4 | **0.091** | **0.010** | 4 |
|  | TSH+ | 0.057 | 0.004 |  | 0.056 | 0.004 |  |
|  | DLK1+/TSH+ | 0.038 | 0.002 |  | 0.035 | 0.002 |  |
|  | *% co-stained DLK1* | *40* |  |  | *38* |  |  |
| FSH+ | **Total proportion** | **0.096** | **0.013** | 2 | **0.092** | **0.000** | 2 |
|  | FSH +ve | 0.077 | 0.011 |  | 0.074 | 0.001 |  |
|  | DLK1+/FSH+ | 0.018 | 0.002 |  | 0.018 | 0.002 |  |
|  | *% co-stained DLK1* | *19* |  |  | *20* |  |  |
| ACTH+ | **Total proportion** | **0.130** | **0.010** | 4 | **0.109** | **0.016** | 4 |
|  | ACTH+ | 0.129 | 0.005 |  | 0.109 | 0.008 |  |
|  | DLK1+/ACTH+ | 0.000 | 0.000 |  | 0.000 | 0.000 |  |
|  | *% co-stained DLK1* | *0* |  |  | *0* |  |  |
|  | **Unclassified** | **0.177** |  |  | **0.213** |  |  |

**Supplementary File 1a**. Proportions of AP cells labelled with hormonal markers in WT and WT-TG female animals at 12 weeks of age.

|  | **Genotype** | **Total body mass (g)** | | |  |  |
| --- | --- | --- | --- | --- | --- | --- |
|  |  | *Mean* | *SD* | *n* | *p vs WT* | *p vs PAT* |
| **E11.5** | WT | 0.0464 | 0.0043 | 11 |  |  |
|  | WT-TG | 0.0458 | 0.0043 | 11 | ns |  |
|  | PAT | 0.0444 | 0.0043 | 8 | ns |  |
|  | PAT-TG | 0.0433 | 0.0081 | 7 | ns | ns |
| **E13.5** | WT | 0.1447 | 0.0136 | 7 |  |  |
|  | WT-TG | 0.1381 | 0.0157 | 17 | ns |  |
|  | PAT | 0.1468 | 0.0111 | 16 | ns |  |
|  | PAT-TG | 0.1464 | 0.0181 | 10 | ns | ns |
| **E18.5** | WT | 1.2062 | 0.1311 | 24 |  |  |
|  | WT-TG | 1.2110 | 0.1060 | 32 | ns |  |
|  | PAT | 1.0205 | 0.0711 | 15 | <0.0001 |  |
|  | PAT-TG | 1.1107 | 0.0808 | 20 | 0.0125 | 0.0513 |
| **P7** | WT | 4.0329 | 0.2682 | 7 |  |  |
|  | WT-TG | 3.8280 | 0.2197 | 5 | ns |  |
|  | PAT | 3.0933 | 0.2542 | 9 | 0.0003 |  |
|  | PAT-TG | 3.7730 | 0.5926 | 10 | ns | 0.0038 |
| **P14** | WT | 7.8124 | 0.6887 | 17 |  |  |
|  | WT-TG | 8.6843 | 0.6171 | 7 | 0.0161 |  |
|  | PAT | 5.6000 | 0.6420 | 8 | <0.0001 |  |
|  | PAT-TG | 6.7640 | 0.2463 | 5 | 0.0097 | 0.0105 |
| **P21** | WT | 10.7120 | 1.6084 | 10 |  |  |
|  | WT-TG | 11.0489 | 1.1016 | 9 | ns |  |
|  | PAT | 8.8117 | 1.0644 | 6 | 0.0129 | ns |
|  | PAT-TG | 10.1354 | 0.7752 | 13 | ns |  |

**Supplementary File 1b**. Total body mass of animals from matched litters sacrificed from E11.5 to P21. Individuals in each age group were compared by One-Way ANOVA with post-hoc pairwise testing WT vs WT-TG, PAT, PAT-TG and PAT vs PAT-TG, corrected for multiple comparisons using Bonferroni's adjustment.

|  | **Genotype** | **AP Volume (mm^3)** | | | | **PP Volume (mm^3)** | | | |  |
| --- | --- | --- | --- | --- | --- | --- | --- | --- | --- | --- |
|  |  | *Mean* | *SD* | *p vs WT* | *p vs PAT* | *Mean* | *SD* | *p vs WT* | *p vs PAT* | *n* |
| **E13.5** | WT | 0.0269 | 0.0056 |  |  | 0.0022 | 0.0005 |  |  | 9 |
|  | WT-TG | 0.0255 | 0.0043 | ns |  | 0.0023 | 0.0004 | ns |  | 11 |
|  | PAT | 0.0164 | 0.0011 | <0.0001 |  | 0.0019 | 0.0002 | ns |  | 6 |
|  | PAT-TG | 0.0172 | 0.0022 | <0.0001 | ns | 0.0021 | 0.0022 | ns | ns | 8 |
| **E18.5** | WT | 0.0600 | 0.0084 |  |  | 0.0092 | 0.0006 |  |  | 7 |
|  | WT-TG | 0.0664 | 0.0059 | ns |  | 0.0096 | 0.0006 | ns |  | 5 |
|  | PAT | 0.0444 | 0.0094 | 0.0025 |  | 0.0102 | 0.0016 | ns |  | 7 |
|  | PAT-TG | 0.0479 | 0.0044 | 0.1670 | ns | 0.0097 | 0.0020 | ns | ns | 8 |

**Supplementary File 1c.** Pituitary volumes acquired by stereological estimation in the embryo. Data from males and females is combined.

|  | **Genotype** | **AL Volume (mm^3)** | | | | **IL Volume (mm^3)** | | | | **PL Volume (mm^3)** | | | |  |
| --- | --- | --- | --- | --- | --- | --- | --- | --- | --- | --- | --- | --- | --- | --- |
|  |  | *Mean* | *SD* | *p vs WT* | *p vs PAT* | *Mean* | *SD* | *p vs WT* | *p vs PAT* | *Mean* | *SD* | *p vs WT* | *p vs PAT* | *n* |
| **P7** | WT | 0.0783 | 0.0069 |  |  | 0.0169 | 0.0020 |  |  | 0.0159 | 0.0022 |  |  | 5 |
|  | WT-TG | 0.0817 | 0.0065 | ns |  | 0.0157 | 0.0019 | ns |  | 0.0165 | 0.0024 | ns |  | 4 |
|  | PAT | 0.0406 | 0.0070 | <0.0001 |  | 0.0110 | 0.0020 | 0.0014 |  | 0.0138 | 0.0036 | ns |  | 8 |
|  | PAT-TG | 0.0565 | 0.0097 | 0.0010 | 0.0074 | 0.0143 | 0.0034 | ns | ns | 0.0157 | 0.0030 | ns | ns | 5 |
| **P14** | WT | 0.1601 | 0.0286 |  |  | 0.0221 | 0.0029 |  |  | 0.0244 | 0.0031 |  |  | 6 |
|  | WT-TG | 0.1795 | 0.0306 | ns |  | 0.0252 | 0.0034 | ns |  | 0.0249 | 0.0044 | ns |  | 7 |
|  | PAT | 0.0857 | 0.0159 | 0.0007 |  | 0.0229 | 0.0018 | ns |  | 0.0168 | 0.0010 | ns |  | 5 |
|  | PAT-TG | 0.0894 | 0.0165 | 0.0046 | ns | 0.0225 | 0.0025 | ns | ns | 0.0196 | 0.0041 | ns | ns | 3 |
| **P21** | WT | 0.1972 | 0.0252 |  |  | 0.0361 | 0.0036 |  |  | 0.0317 | 0.0047 |  |  | 6 |
|  | WT-TG | 0.2617 | 0.0494 | 0.0105 |  | 0.0359 | 0.0076 | 0.0268 |  | 0.0348 | 0.0044 | ns |  | 8 |
|  | PAT | 0.1542 | 0.0043 | ns |  | 0.0301 | 0.0089 | ns |  | 0.0376 | 0.0088 | ns |  | 3 |
|  | PAT-TG | 0.1549 | 0.0257 | ns | ns | 0.0234 | 0.0030 | ns | ns | 0.0251 | 0.0030 | ns | ns | 7 |

**Supplementary File 1d**. Pituitary volumes acquired by stereological estimation in of the intact postnatal gland. Individuals in each age group were compared by One-Way ANOVA with post-hoc pairwise testing WT vs WT-TG, PAT, PAT-TG and PAT vs PAT-TG, corrected for multiple comparisons using Bonferroni's adjustment. Data from males and females is combined.

|  | **Genotype** | **AL Volume (mm^3)** | | | **IL Volume (mm^3)** | | | **PL Volume (mm^3)** | | |  |
| --- | --- | --- | --- | --- | --- | --- | --- | --- | --- | --- | --- |
|  |  | *Mean* | *SD* | *p vs WT* | *Mean* | *SD* | *p vs WT* | *Mean* | *SD* | *p vs WT* | *n* |
| **Males** | WT | 0.570 | 0.081 |  | 0.066 | 0.011 |  | 0.052 | 0.008 |  | 4 |
|  | WT-TG | 0.742 | 0.040 | 0.0126 | 0.080 | 0.014 | 0.4595 | 0.061 | 0.006 | 0.2376 | 3 |
|  | PAT | 0.407 | 0.031 | 0.0195 | 0.046 | 0.007 | 0.1942 | 0.050 | 0.008 | 0.9266 | 3 |
|  | PAT-TG | 0.555 | 0.073 | 0.9789 | 0.067 | 0.021 | 0.9972 | 0.059 | 0.007 | 0.2636 | 6 |
| **Females** | WT | 0.582 | 0.060 |  | 0.059 | 0.019 |  | 0.050 | 0.003 |  | 3 |
|  | WT-TG | 0.837 | 0.151 | 0.0080 | 0.065 | 0.012 | 0.8977 | 0.060 | 0.010 | 0.2003 | 3 |
|  | PAT | 0.509 | 0.021 | 0.4895 | 0.054 | 0.007 | 0.9503 | 0.055 | 0.004 | 0.5488 | 5 |
|  | PAT-TG | 0.404 | 0.034 | 0.0329 | 0.043 | 0.006 | 0.5085 | 0.048 | 0.007 | 0.9841 | 2 |

**Supplementary File 1e**. Pituitary volumes acquired by stereological estimation in of the intact adult gland. All animals were compared by Two-Way ANOVA with post-hoc pairwise testing WT vs WT-TG, PAT, PAT-TG and PAT vs PAT-TG, using Dunnett’s multiple comparison test.

|  | **Genotype** | **CL % p-HH3** | | **NC % p-HH3** | | **Total % p-HH3** | |  |
| --- | --- | --- | --- | --- | --- | --- | --- | --- |
|  |  | *Mean* | *SD* | *Mean* | *SD* | *Mean* | *SD* | *n* |
| **E11.5** | WT | - | - | - | - | 5.59 | 1.04 | 5 |
|  | WT-TG | - | - | - | - | 6.56 | 1.26 | 4 |
|  | PAT | - | - | - | - | 6.80 | 2.89 | 4 |
|  | PAT-TG | - | - | - | - | 7.02 | 2.27 | 4 |
| **E13.5** | WT | 24.34 | 3.42 | 4.01 | 1.91 | 11.77 | 3.09 | 7 |
|  | WT-TG | 24.29 | 2.68 | 4.71 | 1.29 | 12.49 | 2.88 | 8 |
|  | PAT | 19.89 | 2.28 | 3.53 | 1.04 | 9.69 | 0.85 | 7 |
|  | PAT-TG | 18.85 | 2.48 | 2.95 | 0.78 | 9.06 | 1.36 | 8 |
| **E18.5** | WT | 10.34 | 1.43 | 8.46 | 0.43 | 8.94 | 0.30 | 5 |
|  | WT-TG | 10.56 | 2.26 | 8.42 | 2.10 | 8.97 | 2.00 | 7 |
|  | PAT | 10.64 | 2.91 | 8.76 | 3.66 | 9.25 | 3.42 | 8 |
|  | PAT-TG | 11.19 | 2.86 | 11.02 | 3.50 | 11.01 | 3.20 | 8 |
| **P7** | WT | 12.71 | 1.22 | 4.58 | 1.63 | 5.15 | 1.59 | 6 |
|  | WT-TG | 5.73 | 1.15 | 2.33 | 0.25 | 2.55 | 0.29 | 3 |
|  | PAT | 12.91 | 3.91 | 7.17 | 0.73 | 7.59 | 0.86 | 6 |
|  | PAT-TG | 11.87 | 4.22 | 5.50 | 0.90 | 5.94 | 0.63 | 4 |
| **P14** | WT | 3.92 | 1.22 | 9.18 | 1.25 | 8.91 | 1.24 | 4 |
|  | WT-TG | 5.56 | 2.11 | 11.32 | 0.95 | 11.52 | 0.87 | 5 |
|  | PAT | 4.54 | 1.51 | 7.70 | 0.79 | 7.66 | 0.87 | 3 |
|  | PAT-TG | 4.76 | nd | 6.97 | nd | 6.87 | nd | 1 |
| **P21** | WT | 5.66 | 1.09 | 1.57 | 0.22 | 1.82 | 0.25 | 3 |
|  | WT-TG | 7.35 | 0.36 | 1.65 | 0.49 | 2.07 | 0.47 | 3 |
|  | PAT | 6.01 | 1.44 | 1.81 | 0.15 | 2.04 | 0.20 | 2 |
|  | PAT-TG | 6.52 | 1.58 | 2.42 | 0.37 | 2.70 | 0.46 | 3 |

**Supplementary File 1f**. Proportion of proliferating cells (IHC positive for p-HH3) in the embryonic and postnatal pituitary gland. Data from males and females is combined.

| **Probe target** | **Cat#** | **Source** |
| --- | --- | --- |
| Mm-Lef1 | 441861 | ACDBio |
| Mm-Axin2 | 400331 | ACDBio |
| Mm-Shh | 314361 | ACDBio |
| Mm-Fgf8 | 313411 | ACDBio |
| Mm-Fgf10 | 446371 | ACDBio |

**Supplementary File 1g.** RNAScope probes used in this study

| **Antibody target (species)** | **Source** | **Antibody titre** |
| --- | --- | --- |
| DLK1 (mouse, WB) | Abcam ab21682 | 1:500 |
| DLK1 (mouse, IHC) | R&D AF8277 | 1:200 |
| DLK1 (mouse, IHC) | Abcam ab210471 | 1:1000 |
| Alpha tubulin (human, WB) | Merck-SIGMA T5168 | 1:10,000 |
| GH (Rat) | National Hormone and Peptide Program (NHPP) | 1:1000 |
| PRL (Mouse) |  | 1:500 |
| TSHβ (Rat) |  | 1:500 |
| FSHβ (Rat) |  | 1:500 |
| ACTH (Rat) |  | 1:1000 |
| LH (Rat) |  | 1:500 |
| POU1F1 (Mouse) | A gift from S. Rhodes, Indiana University School of Medicine, Indianapolis USA | 1:300 |
| SOX2 (Mouse) | Abcam ab92494 | 1:400 |
| α-phospho-Histone H3-Ser10 (human) | Merck-SIGMA 06-570 | 1:300 |
| HES1 (human) | Cell Signaling Technologies D6P2U | 1:300 |
| Goat SOX2 | R&D AF2018 | 1:300 |

**Supplementary File 1h.** Primary antibodies used in the study.

| **Target** | **Fw** | **Rv** |
| --- | --- | --- |
| Dlk1 qPCR | GAAAGGACTGCCAGCACAAG | CACAGAAGTTGCCTGAGAAGC |
| Dlk1 splice | CTGCACACCTGGGTTCTCTG | TCCTCATCACCAGCCTCCTT |
| Ghrh | GCTGTATGCCCGGAAAAGTGAT | AATCCCTGCAAGATGCTCTCC |
| Sst | CCCAGACTCCGTCAGTTTCT | GGGCATCATTCTCTGTCTGG |
| Actb | TTCTTTGCAGCTCCTTCGTT | ATGGAGGGGAATACAGCCC |
| Tuba | AGACCATTGGGGGAGGAGAT | GTGGGTTCCAGGTCTACGAA |

**Supplementary File 1i.** RT-PCR and RT-qPCR primers used in this study.
